# Potassium Channels and Their Potential Roles in Substance Use Disorders

**DOI:** 10.3390/ijms22031249

**Published:** 2021-01-27

**Authors:** Michael T. McCoy, Subramaniam Jayanthi, Jean Lud Cadet

**Affiliations:** Molecular Neuropsychiatry Research Branch, NIDA Intramural Research Program, Baltimore, MD 21224, USA; mmccoy@intra.nida.nih.gov (M.T.M.); sjayanth@intra.nida.nih.gov (S.J.)

**Keywords:** alcohol, cocaine, methamphetamine, opioids, pharmacotherapy

## Abstract

Substance use disorders (SUDs) are ubiquitous throughout the world. However, much remains to be done to develop pharmacotherapies that are very efficacious because the focus has been mostly on using dopaminergic agents or opioid agonists. Herein we discuss the potential of using potassium channel activators in SUD treatment because evidence has accumulated to support a role of these channels in the effects of rewarding drugs. Potassium channels regulate neuronal action potential via effects on threshold, burst firing, and firing frequency. They are located in brain regions identified as important for the behavioral responses to rewarding drugs. In addition, their expression profiles are influenced by administration of rewarding substances. Genetic studies have also implicated variants in genes that encode potassium channels. Importantly, administration of potassium agonists have been shown to reduce alcohol intake and to augment the behavioral effects of opioid drugs. Potassium channel expression is also increased in animals with reduced intake of methamphetamine. Together, these results support the idea of further investing in studies that focus on elucidating the role of potassium channels as targets for therapeutic interventions against SUDs.

## 1. Introduction

Substance use disorders (SUDs) are biopsychosocial disorders include neuropsychiatric symptoms such as loss of control of drug taking, repeated relapses to drug taking after intervals of forced or voluntary abstinence, and continued drug use in the presence of adverse consequences as described in the Diagnostic Statistical Manual of the American Psychiatric Association [1]. Individuals who use licit and illicit drugs, including alcohol, cocaine, methamphetamine, or opioids, do not all develop SUDs because of genetic and environment factors that enable them to be resilient [2]. On the other hand, several approaches have been taken to develop treatment approaches to alter the clinical course of SUDs in those patients who meet diagnostic criteria and subsequently seek medical and psychological treatment [3,4,5]. These approaches have met with variable degrees of success. However, there still remains a substantial need to develop pharmacotherapies that can provide important relief for individuals who have progressed from the use of these drugs for their reward properties to their compulsive abuse even in the presence of adverse medical and social consequences. Thus, the goal of the present review is to discuss the possibility of targeting potassium channels as potential pharmacological treatment for SUDs.

### 1.1. Classification of Potassium Channels

Ion channels are integral pore-forming transmembrane proteins that selectively control the influx and efflux of important physiological ions including Na^+^, K^+^, Ca2^+^, and Cl^-^ into and from cells or intracellular organelles. They serve to control cytoplasmic and intraorganellar concentrations of these ions as well as regulate membrane potential and cell volume. Ion channels including potassium channels participate in various cellular processes and physiological events that include action potential propagation and neurotransmission. These channels participate in the regulation of thinking and memory processes as well as other brain functions [6]. Abnormalities in these channels appear to be involved in several diseases that affect the central nervous system (CNS) including seizure disorders [7,8]. Therefore, it is not farfetched to suggest these ion channels may be promising drug targets for the development of novel therapeutics. 

The separation of the sodium and K^+^ currents were first described in squid neurons [9]. The cloning of the first voltage-gated K^+^ channel, Shaker gene, was published by [10] using Drosophila. This description was followed by the discovery of many more K^+^ channels [11]. Ninety identified K^+^ channels have been divided into subgroups based on their molecular structures and mechanisms of activation [12,13]. These include (1) voltage-gated (K_V_), (2) calcium-activated (K_Ca_), (3) two/tandem-pore domain (K_2P_) subgroup, (4) inwardly rectifying (K_IR_) subgroup. Table 1 summarizes the classification of potassium channels.

The largest subgroup of potassium channels consists of voltage-gated (K_V_) channels that include 42 genes in 11 families. The K_V_ family is fine-tuned by subunit compositions (homo- or hetero –polypeptides), their localization, and voltage threshold in the cell [14]. Voltage-gated K^+^ channels detect voltage changes by the voltage sensor domain that is coupled to the pore-gate domain [15]. The majority of Kv family genes (K_V_1–K_V_12) have been found in the brain [16], where they are involved in neuronal processes responsible for the variation of axonal and dendritic action potentials via its effects on threshold, burst firing, and firing frequency [11]. KCNQ channels (Kv7) are voltage-dependent potassium channels composed of five KCNQ subunits (KCNQ1–5 or Kv7.1–7.5). 

The heterogeneous family of calcium-activated (K_Ca_) channels depends on changes in intracellular Ca^2+^ concentrations to control membrane potentials and cellular excitability while maintaining K^+^ homeostasis [17,18]. The K_Ca_ channel is represented by 12 genes in 3 families that are (1) small-conductance Ca^2+^-activated (KCNN1-3), (2) large-conductance Ca^2+^- and voltage-activated (KCNMA1, KCNB1-4) and (3) sodium-activated (KCNT1-2, KCNU1). Large conductance Ca^2+^- and voltage-activated K^+^ channel (K_Ca_1.1, KCNMA1) monomer has two modules, one for voltage-sensing and another that is co-localized with L-type Ca^2+^ channels [19,20]. In addition, auxiliary subunits KCNMB1-4 co-assembly and other factors changes channel selectivity including pharmacological properties [21,22]. Ca^2+^ currents (N-type) have been shown to regulate neurotransmitter release in patch clamp studies [23]. Small conductance (SK) K_Ca_ channels in comparison to BK channels run at a slower pace and are gated solely by intracellular concentration of calcium ions. Activation of SK channels leads to the efflux of K^+^ ions shifting of the membrane potential to a more negative environment, with generation of a long-lasting hyperpolarization (See Bond et al., for an overview) [24].

The members of the two/tandem-pore domain (K_2P_) subgroup are also known as ‘‘leak’’ or ‘‘background’’ K^+^ (IK_leak_) channels. The regulation of K_2P_ channels is complex and their functions are very important in the CNS [25]. K_2P_ channels participate in the regulation of resting membrane potentials, action potential duration, and modification of the sensitivity of cells to synaptic inputs [26,27,28]. K_2P_ channels (K_2P_1–K_2P_18) include 15 genes subdivided into 6 families named TREK, TASK, TRESK, TWIK, THIK, and TALK [25,29,30]. TREK channels are mechano-sensitive while TASK channels (KCNK3, KCNK5, KCNK9, KCNK15, KCNK17) are acid pH-sensitive. TWIK channels (KCNK1, KCNK6, KCNK7) are acid-sensitive and weak inwardly rectifying. THIK channels (KCNK12, KCNK13) are TWIK-related halothane-inhibited K^+^ channels. The TALK channel (KCNK16) is alkaline pH-sensitive. 

The inwardly rectifying (K_IR_) subgroup, named after its inward flow of K^+^ ions, was first cloned in 1993 [31]. These channels are arranged into homo- or hetero-tetramers that react to magnesium and polyamines [32]. This group contains 16 genes (K_IR_1–K_IR_7) that are divided into 4 families: K^+^ transport channels (KCNJ1, KCNJ10, KCNJ13, KCNJ15); classical K_IR_ channels (KCNJ2, KCNJ4, KCNJ12, KCNJ14, KCNJ16, KCNJ18); G-protein K^+^ channels (KCNJ3, KCNJ5, KCNJ6, KCNJ9); ATP-sensitive K^+^ channels (KCNJ8, KCNJ11). 

### 1.2. K^+^ Channels and Neuronal Function

Potassium channels perform multiple functions in the neuron. These include maintaining resting cell membrane potential, modulating neuronal excitability, neurotransmitter release, and maintaining homeostasis [11,33,34]. The sub-cellular localization of K^+^ channels in axon terminals [35] provides a mechanism via which they can control cellular communication via dopamine (DA) neurotransmission in reward circuitries [36,37,38].

In fact, KCNQ2 and KCNQ3 channels that are expressed in the brain as hetero-tetramers (KCNQ2/3) [39] are localized on dopamine neurons in the ventral tegmental area (VTA) [37].

Activation of KCNQ2/3 has been shown to reduce midbrain dopamine neuronal excitability and to also attenuate psychostimulant-induced increases in extracellular dopamine in the nucleus accumbens [40,41,42]. Activation of KCNQ2/3 was shown to reduce the firing of midbrain dopaminergic neurons, to inhibit striatal dopamine synthesis [40]. Retigabine, another KCNQ activator, was also reported to prevent d-amphetamine-induced DA efflux in the nucleus accumbens and d-amphetamine-induced locomotor hyperactivity [42]. Similar results have been obtained using striatal slices whereby retigabine was able to inhibit KCl-dependent release of DA [43]. Together these results implicate potassium in the regulation of DA release and stimulant-induced behavioral activation.

Their widespread localization in brain regions that regulate decision making (prefrontal cortex—PFC), reward (nucleus accumbens—NAc and midbrain), learning and memory (hippocampus—HIP) supports a role of K^+^ channels in the acquisition and/or maintenance of drug taking behaviors [34,44,45,46,47].

The accumulated evidence suggests that K^+^ channels can regulate and/or facilitate the development of neuroadaptations that may be important in drug-induced progression of behaviors that are associated with the development of a SUD diagnosis [48]. Indeed, K^+^ channel signaling appears to be impacted by exposure to various rewarding drugs that include alcohol [49,50,51], cocaine [52], methamphetamine [53,54,55], and opioids [56,57], morphine [58,59], oxycodone [60], and fentanyl [61]. Detailed effects of these drugs on potassium channels are described below.

## 2. Potassium Channels and Alcohol Use Disorder

Alcohol consumption is widespread in the general population in the world [62,63]. However, although relatively few individuals meet criteria for alcohol use disorder (AUD) [64], AUD is associated with significant medical, neurological, and psychiatric comorbidities with consequent increased morbidity and mortality [65]. Presently, there are few FDA-approved pharmacological treatments for AUD. These include disulfiram, acamprosate, and naltrexone and these medications have limited success at reducing the high rates of relapse and are not necessarily efficacious in some populations [66,67]. Thus, there is a need to develop other pharmacological agents based on the elucidation of neurobiological mechanisms that might be the substrates of AUD. 

Herein, we have reported both the causal and non-causal role of K^+^ channels in AUD. Studies that show changes in gene expression should be considered descriptive and of need for further validation. However, data from pharmacological interventions and knock-out animal models should be considered as having shown potential causal relationships. High throughput microarray analyses using rodent models of AUD have found differential expression of genes that encode K^+^ channels in the brain of animals with different levels of alcohol intake [68,69]. Significantly, FDA-approved potassium channel regulators reduce alcohol intake in rats [70].

### 2.1. Alcohol Use Disorder and K_V_ Family

Rinker et al. (2017) identified significant changes in the expression of several genes that belong to K_V_ families in the PFC and NAc of mice that showed different levels of alcohol intake [68]. In the PFC, they reported that voluntary ethanol consumption (VEC) results in changes in K*cna5*, K*cnc3*, K*cnd2*, and K*cnq5* expression [68]. Chronic intermittent ethanol (CIE) caused changes in the expression of K*cnb1*, K*cnc*1, and K*cnq5* whereas heavy alcohol use (HAU) impacted K*cna7*, K*cnc1*, K*cnd2*, K*cne1*, and K*cnh3* levels. In the NAc, voluntary ethanol consumption induced changes in K*cna1*, K*cnab3*, K*cnb1*, K*cnd2*, K*cne1*, K*cnq2*, and K*cnq5* mRNA levels. CIE exposure influenced the expression of K*cnb1*, K*cnc2*, K*cnc4*, K*cnd2*, and K*cnv1* whereas heavy alcohol use impacted K*cna4*, K*cnh2*, K*cnh7*, K*cnq1*, K*cnq5*, K*cns1*, K*cns2*, and K*cnv2* expression (Table 2) [68]. These observations are, in part, consistent with a previous report that chronic ethanol exposure caused downregulation of K*cnq2* mRNA expression was observed in the mouse amygdala [71]. Another K_V_ channel, K_V_4.2/KCND2, has been reported to show alcohol exposure-induced decreases in protein levels with increasing concentrations of alcohol in both in-vitro and in-vivo model [72,73]. These results are consistent with the report that administration of retigabine, an activator of K_V_7.2/KCNQ2 and K_V_7.3/KCNQ3, led to a reduction of voluntary alcohol intake in rodents [74,75]. It is to be noted that retigabine has been withdrawn from the market by its manufacturer GlaxoSmithKline.

### 2.2. AUD and K_Ca_ Families

K_Ca_ (Ca^2+^-activated) channels are targets for both acute and chronic alcohol exposure [70,82,83,84,85]. Large (BK) and small conductance (SK) K_Ca_ channels influence cell repolarization and impact dendritic Ca^2+^ spikes [84]. Rinker et al. (2017), using their high-throughput assay following different levels of ethanol exposure, have reported significant changes in BK channel expression of K*cnma1* and K*cnmb2*, in both PFC and NAc of mice [68]. In addition to changes in mRNA expression, acute ethanol administration induced significant decreased KCNMA1 protein levels in primary hippocampal neuronal cultures [82]. Marrero et al. (2015) reported that moderate drinkers did not exhibit changes in KCNMA1 expression whereas heavy drinkers showed decreased KCNMA1 expression [86].

Unlike, BK channels whose levels are changed with acute ethanol exposure, the expression of SK (KCNN) channels is influenced by chronic ethanol administration, with reduced expression of K_Ca_2.3/K*cnn3* channels and KCNN3 protein levels being observed in the rodent NAc [79]. Interestingly, protracted withdrawal from chronic ethanol reduced the function and trafficking of KCNN3 in the rodent NAc [78].

### 2.3. AUD and K_2P_ Families

Members of K_2P_ subfamilies are also differentially regulated by alcohol intake [50,80]. Specifically, K_2P_7.1/K*cnk13*/T*hik1* mRNA expression in the mouse ventral tegmental area (VTA) was upregulated by acute ethanol exposure [50] whereas mRNA levels of K_2P_1.1/K*cnk1*/T*wik1*, a weak inward rectifying channel, were down-regulated in the mouse cerebellum by chronic ethanol exposure [80]. Those results are interesting because Twik1 functions to set the resting membrane potential in cerebellar cells [87]. Interestingly, chronic nicotine exposure also down-regulated T*wik1* expression in the amygdala [88], suggesting the possibility that Twik1 might participate in the long-term effects of rewarding drugs on the brain. Genome-wide studies have suggested the possibility that KCNK2 gene might be linked to AUD in the native Indians and the Euro-Americans [89].

Potassium channels might also play a role in the fetal alcohol syndrome. Specifically, Ramadoss et al. (2008) infused pregnant ewes with ethanol or saline using a “3 days/week binge” pattern during the third trimester [90]. They reported that chronic ethanol causes a 45% reduction in the total number of fetal cerebellar Purkinje cells. They also found that TASK1 channels were expressed in Purkinje cells and that the TASK3 isoform was expressed in granule cells of the ovine fetal cerebellum. Importantly, pharmacological blockade of both TASK1 and TASK3 channels simultaneous with ethanol was able to prevent the reduction in fetal cerebellar Purkinje cell number [90].

### 2.4. AUD and K_IR_ Families

G protein-gated inwardly rectifying potassium (K_IR_) channels assemble into heterotetramers of K_IR_3.1/3.2/Kcnj3/6, K_IR_3.1/3.3/Kcnj3/9, K_IR_3.1/3.4/Kcnj3/5, or K_IR_ 3.2/3.3/Kcnj6/9 subunits or in some cases homotetramers of Kcnj6 subunits [91]. K_IR_ channels regulate neuronal excitability and can be activated by ethanol. It is to be noted here that all K_IR_ channels are not affected by alcohol exposure. For example, K_IR_.2.1-.2.4/Kcnj2/14 and K_IR_1.1/Kcnj1 channels are unaffected by alcohol consumption, while K_IR_3.1-3.4 channels are activated [91,92]. Surprisingly, ifenprodil was shown to block Kcnj3/5 higher than Kcnj3/6 and Kcnj6 homotetramers while K_IR_.2.1-.2.4/Kcnj2/14 and K_IR_1.1/Kcnj1 channels are insensitive [93].

The study by Herman et al. (2015) exhibits the role of K_IR_ 3.3/Kcnj9 as a critical gatekeeper of ethanol incentive salience and projects as a potential target for the treatment of excessive ethanol consumption [81]. Similar to Herman et al. (2015), knock-out (KO) of K_IR_3.3/KCNJ9/protein enhanced ethanol conditioned place preference [94,95].

Rinker et al. (2017) also identified several K_IR_ genes, in addition to K_V_, K_Ca_ and K_2P_ genes in all the three paradigms of alcohol intake [68] (voluntary, CIE and heavy use—see Table 2). Interestingly, the major gene expression changes were identified in the NAc.

## 3. Potassium Channels and Cocaine Use Disorder

In recent years, overdose death related to cocaine use disorder (CUD) is on the rise and have almost tripled in the US [96]. Amongst the US population of cocaine users, about 5.5 million (2.0% of the population) were above the age of 12 and about 2.2% of high school seniors used cocaine [97]. Cocaine exerts its actions by blocking the dopamine (DA) transporter, with secondary increases in DA in the synaptic cleft [98]. These facts have led to therapeutic approaches that include the development of anti-DAergic drugs in attempts to treat CUD. These approaches have not met with a high degree of success [99,100], suggesting there are potential targets that might offer other avenues to fight cocaine addiction.

Recent studies have indeed suggested a causal role for potassium channels in CUD. Specifically, Mooney & Rawls, (2017) reported that flupirtine, an agonist of the voltage-gated KCNQ2/3 channel, can reduce the development of cocaine place preference and locomotor activation [101]. Studies using knock-out mice models have reported that K*cnj6* and K*cnj9* knockout mice exhibited reduced cocaine seeking compared to WT mice [102]. However, only K*cnj6* knock-out mice displayed increased locomotion behavior [102]. In contrast, McCall et al., (2017) reports increased cocaine self-administration in mice with K*cnj6* deletion in VTA DA neurons [52]. The discrepancy observed between these two studies may be due to the way that the gene was deleted. The former uses whole animal KO whereas the latter use site-specific ablation. In addition, K*cnj6* ablation is known to play a key in the regulation of K_IR_3/GIRK channel activity in midbrain DA neurons [103,104] (See Table 3).

## 4. Potassium Channels and Methamphetamine Use Disorder

METH use disorder (MUD) is highly prevalent throughout the world and characterized by loss of control over drug use despite adverse consequences as well as strong urges to seek and use the drug [1]. Cognitive and psychiatric deficits consequent to structural and functional pathologies have been well documented [105,106].

Researchers in the Cadet laboratory have recently published several papers in which they attempt to mimic human MUD conditions in rodent models [53,55,107,108,109]. In those models, they have introduced an additional DSM5 criterion that is related to compulsive drug intake despite adverse consequences [107]. In that model, they use drug self-administration and then introduced footshock punishment to represent adverse consequences that humans might encounter during their frequent experiences with drugs. In the case of METH, shocks are introduced after the animals had escalated their intake and reached a plateau of consistent daily consumption of large amount of METH. Our group has shown that the introduction of footshocks administered contingently with METH can help to dichotomize rats into punishment or shock-resistant (compulsive, vulnerable to addiction, addicted) and shock-sensitive (not vulnerable, non-addicted) animals [107,109,110,111]. In addition to the behavioral manifestations, the resistant and sensitive rats have also been shown to exhibited interesting transcriptional and biochemical changes in certain brain regions including the dorsal striatum and nucleus accumbens [107,110,112]. It is to be noted here that unlike alcohol and cocaine, studies with METH have focused on non-causal association with potassium channels and requires further elucidation.

### 4.1. MUD and K_V_ Family

Recently, we used that model to investigate potential alterations in global DNA hydroxymethylation in the nucleus accumbens (NAc). We used the NAc because behavioral phenomena that occur METH self-administration (METH SA) are thought to be regulated by interconnections between distinct brain regions that include that structure [113]. Neuroplastic changes in the NAc are thought to participate in the development and maintenance of drug-taking behaviors [114]. We found, for the first time, that rats that continued to take METH compulsively exhibited differential DNA hydroxymethylation in comparison with both control and nonaddicted rats [55]. Rats that suppress their intake of METH in the presence of footshocks also showed differences in DNA hydroxymethylation from control rats, suggesting that exposure to the drug can result in prolonged effects on this epigenetic marker. The changes in DNA hydroxymethylation in the NAc of non-addicted rats were observed mostly at intergenic sites located on long and short interspersed elements. Of significant relevance to the present review, we also observed differentially hydroxymethylated regions in genes that encoded voltage (K_V_1.1, K_V_1.2, K_Vb_1 and K_V_2.2) potassium channels [55]. In order to test if these changes in DNA hydroxymethylation were accompanied with changes in transcription, we used quantitative PCR and measured their mRNA levels in the experimental groups of rats. We found, indeed, that the mRNA levels of these potassium channels were increased in the non-addiction rats. These results suggest that increased expression of these channels might participate in suppressing METH self-administration by rats.

The potential role of potassium channels in METH taking behaviors was also investigated in a subsequent paper. In that paper, we tested the possibility that a single pre-exposure to METH could potentiate its intake during self-administration experiments [53]. Animals were therefore pre-treated with saline or METH prior to METH SA. The experiment consisted of three experimental groups: (1) a single saline injection followed by saline self-administration (SS); (2) a single saline injection followed by METH SA (SM); and (3) a single METH injection followed by METH SA (MM). METH-pretreated rats escalated METH SA earlier and took more METH than saline-pretreated animals. Because compulsive METH takers and METH-abstinent rats show differences in potassium (K^+^) channel mRNA levels in their nucleus accumbens (NAc), we tested the possibility that the expression of K_V_ potassium channels might also help to distinguish between rats that escalated METH earlier (MM group) than the other group (SM). Increased levels of mRNA and protein expression of voltage-gated K+ channels (Kv1: Kcna1, Kcna3, and Kcna6) were indeed found in the NAc of rats that escalated METH later and took less METH [53]. Rats with increased mRNA expression also showed decreased DNA methylation at the CpG-rich sites near the promoter region of these genes.

It is to be noted that potassium channels may also be involved in the toxic effects [115] that have been reported after various doses of METH [116]. Specifically, Zhu et al., (2018) treated primary cultured hippocampal neurons with METH and reported that the drug caused time- and dose-dependent increases in K_V_2.1 protein expression which was accompanied by elevated cleaved-caspase 3 and declined bcl-2/bax ratio, markers of neuronal apoptosis as previously reported after METH injections into rats [117]. Blockage of K_V_2.1 with the inhibitor, GxTx-1E, or its knockdown attenuated the toxic effects of the drug [116].

### 4.2. MUD and K_Ca_ Family

The investigators from Cadet’s laboratory also documented changes in DNA hydroxymethylation in genes that encode K_Ca_ potassium channel genes in the NAc of animals exposed to METH and subsequently dichotomized by footshocks [55]. These genes included K_Ca_2.1/KCNN1 and K_Ca_2.2/KCNN2. Similar to the observations for K_V_ channels, these changes were associated with increased expression of their mRNA levels. We also investigated the expression of calcium-activated K^+^ channels in animals that had received saline or METH prior to being put through the METH SA experiments [53]. Interestingly, only K*cnn1* (K_Ca_2.1) showed increased expression in the NAc of rats that received saline first (SM rats) in comparison to those that received an injection of METH first (MM rats). In contrast, *Kcnn3* (KCa2.3/SKCa3) and *Kcnma1* mRNA levels were increased in all rats that had self-administered METH [53], suggesting that METH SA is enough to alter the expression of these genes irrespective of the amount of METH taken. (See Table 4).

## 5. Potassium Channels and Opioid Use Disorders

Opioid use disorders (OUDs) have reached epidemic levels [117]. The number of opioid-related overdose deaths from both prescription and illicit opioids reached close to 450,000 between 1999–2018 [118]. In 2018 alone, 46,802 people died of opioid overdoses, representing 69.5% of all drug overdose deaths [119]. Some of these terrible consequences might be related to treatment availability which is quite limited. Presently, FDA-approved medications for OUD include the mu-opioid receptor full agonist methadone, mu-opioid receptor partial agonist buprenorphine, and mu-opioid receptor antagonist naltrexone [120]. There are therefore windows for the development of additional medications that may be derived from a basic understanding of the effects of opioid drugs.

### 5.1. OUDs and K_V_ Family

There is causal evidence that potassium channels might be involved in the effects of opioid drugs, suggesting the possibility that activators of potassium channels might be useful in the treatment of pain and/or opioid addiction [121]. For example, a genome-wide association study using opioid-dependent humans has identified a risk variant in the voltage-gated K+ channel gene K_V_6.2/K*cng2* [57]. In addition, the use of selective K_V_7/KCNQ2 -3 K+ channel activator, flupirtine, increased the analgesic effects of morphine in animal pain models [122,123]. Moreover, another KCNQ K^+^ channel activator, retigabine/ezogabine, elevated pain threshold and prolonged withdrawal latency after thermal stimulation [124]. Interestingly, the synergistic analgesic effect observed may eventually translate to reduced opioid prescription. The effects of retigabine could be blocked by the potassium channel antagonist, linopirdine [124]. In-vitro studies have shown the effects of opioid agonists, fentanyl, heroin, and methadone, on voltage-dependent potassium channels, K_V_11.1/KCNH2/hERG [61,125,126]. It needs to be emphasized that the pharmacological activation of cardiac hERG potassium channels have adverse effects and warrants caution in clinical research. (see Table 5).

### 5.2. OUDs and K_Ca_ Family

Knock-down of BK channel, *Kcnb3* via small interference RNA was reported to ameliorate the hyperalgesia and anti-nociceptive effects of chronic morphine [128]. In addition to the big conductance K^+^ channel, the small conductance SK channel also plays a specific role in reward circuits following morphine exposure. Specifically, Fakira et al. (2014) treated mice with escalating doses of morphine over 4 days followed by a challenge dose of morphine a week later. The dosing morphine schedule resulted in increased locomotor activity and increased SK2 activation [127]. The animals that received repeated morphine followed by a morphine challenge exhibited increased expression of SK2 protein levels in whole hippocampal homogenates but decreased SK2 expression in post-synaptic density [127]. A more recent study also provided evidence for the involvement of SK channels in the actions of opioid drugs [58]. The authors sought to determine if SK channels located in the nucleus accumbens played any role in morphine withdrawal. They found that firing of neurons in the shell of the nucleus accumbens was enhanced secondary to decreased expression of SK channels during morphine withdrawal, with SK2 and SK3 protein levels being significantly decreases after 3 weeks of withdrawal [58]. These observations support a role of potassium channels in the longterm effects of morphine in the brain.

### 5.3. OUDs and K_IR_ Family

Opioids also exert significant effects on KIR family channels. These have been reported on the G-protein gated class that includes GIRK1-4/KCNJ3, 5, 6 and 9. The analgesic effects of morphine were potentiated in knock-out mice with Kcnj6/ GIRK2/ K_IR_3.2 deletion [129]. Similarly, the antinociceptive effects of oxycodone were attenuated by knock-down of K*cnj3*/ GIRK1/ K_IR_3.1channels using short interfering RNA [60]. Studies by Kotecki et al. (2015) using K*cnj3* and K*cnj6* knock-out mice models have shown that enhancement of morphine-induced locomotion.

Blockers of ATP-sensitive class of K_IR_ subfamily, glibenclamide and tolbutamide sulfonylureas, effectively reversed the peripheral antinociceptive effect of fentanyl [130]. Glibenclamide was also shown to play an active role in morphine reward [131] by facilitating morphine-induced conditioned place preference [132].

## 6. Conclusions

This review has provided descriptive and causal evidence for diverse roles of K^+^ channels in the behavioral manifestation of substances of abuse including alcohol, cocaine, methamphetamine, and opioids. These substances can alter the expression of potassium channels at both mRNA and protein levels. These changes were found to occur in various brain regions including the nucleus accumbens and hippocampus that are involved in various aspects of addiction. In addition, we reviewed, evidence that activators of K^+^ channels can suppress behaviors induced by some of these rewarding agents. These behaviors include drug acquisition, maintenance, and withdrawal-associated phenomena. The evidence discussed herein supports the view that there is a need to invest in the development of pharmacotherapeutic agents that target K^+^ channels in the brain. These studies will help to develop non-dopaminergic agents against SUDs since DAergic drugs have not been shown to be very efficacious against these psychiatric disorders.

## Figures and Tables

**Table 1:** Families of Potassium Channels.

Voltage-Gated			Calcium/Sodium-Activated			Two-Pore Domain			Inwardly Rectifying		
K_V_ 1	Shaker-related	KCNA1-10	*Large-conductance Ca^+^-activated*			*TREK*			*K^+^-transport*		
K_V_ 2	Shab-related	KCNB1-2	K_Ca_ 1.1	BKα	KCNMA1	K_2p_ 2.1	TREK1	KCNK2	K_IR_ 1.1	ROMK	KCNJ1
K_V_ 3	Shaw-related	KCNC1-4		BKβ	KCNMB1-4	K_2p_ 10.1	TREK2	KCNK10	K_IR_ 4.1	BIRK1	KCNJ10
K_V_ 4	Shal-related	KCND1-3	*Small-conductance Ca^+^-activated*			*TASK*			K_IR_ 7.1	LCA16	KCNJ13
K_V_ 5		KCNF1	K_Ca_ 2.1	SK1	KCNN1	K_2p_ 3.1	TASK1	KCNK3	K_IR_ 4.2	IRKK	KCNJ15
K_V_ 6		KCNG1-4	K_Ca_ 2.2	SK2	KCNN2	K_2p_ 5.1	TASK2	KCNK5	*Classical*		
K_V_ 7		KCNQ1-5	K_Ca_ 2.3	SK3	KCNN3	K_2p_ 9.1	TASK3	KCNK9	K_IR_ 2.1	IRK1	KCNJ2
K_V_ 8		KCNV1-2	K_Ca_ 3.1	SK3	KCNN4	K_2p_ 15.1	TASK5	KCNK15	K_IR_ 2.3	IRK3	KCNJ4
K_V_ 9		KCNS1-3	*Na^+^-activated*			K_2p_ 17.1	TASK4	KCNK17	K_IR_ 2.2	IRK2	KCNJ12
K_V_ 10.1	EAG1	KCNH1	K_Ca_ 4.1	Slo2.2	KCNT1	*TRESK*			K_IR_ 2.4	IRK4	KCNJ14
K_V_ 10.2	EAG2	KCNH5	/ K_NA_ 1.1			K_2p_ 18.1	TRESK1	KCNK18	K_IR_ 5.1	BIR9	KCNJ16
K_V_ 11.1	HERG1	KCNH2	K_Ca_ 4.2	Slo2.1	KCNT2	*TWIK*			K_IR_ 2.6	TTPP2	KCNJ18
K_V_ 11.2	HERG2	KCNH6	/ K_NA_ 1.2			K_2p_ 1.1	TWIK1	KCNK1	*G-protein gated*		
K_V_ 11.3	HERG3	KCNH7	K_Ca_ 5.1	Slo3	KCNU1	K_2p_ 6.1	TWIK2	KCNK6	K_IR_ 3.1	GIRK1	KCNJ3
K_V_ 12.1	ELK1	KCNH8				K_2p_ 7.1	TWIK3	KCNK7	K_IR_ 3.4	GIRK4	KCNJ5
K_V_ 12.2	ELK2	KCNH3				*THIK*			K_IR_ 3.2	GIRK2	KCNJ6
K_V_ 12.3	ELK1	KCNH4				K_2p_ 12.1	THIK2	KCNK12	K_IR_ 3.3	GIRK3	KCNJ9
						K_2p_ 13.1	THIK1	KCNK13	*ATP-sensitive*		
						*TALK*			K_IR_ 6.1	uKATP1	KCNJ8
						K_2p_ 16.1	TALK1	KCNK16	K_IR_ 6.2	IKATP	KCNJ11
						*TRAAK*					
						K_2p_ 4.1		KCNK4			

**Table 2:** Potassium Channels Effected by Alcohol.

Species/Source	Experimental Design	Comparison	Voltage-Gated	Calcium-activated	Two-Pore domain	Inwardly Rectifying	Ref.
Mouse, Nac, PFC	BXD, EtOH treatment, Array	VEC, NAc	*Kcna1, Kcnab3, Kcnb1, Kcnd2, Kcne1, Kcnq2, Kcnq5, and Kcnv1*	*Kcnma1, Kcnn1, Kcnn3, and kcnt1*	*Kcnk4*	*Kcnj3, and Kcnj15*	[68]
		VEC, PFC	*Kcna5, Kcnc3, Kcnd2, Kcnq5, and Kcns1*	*Kcnma1*		*Kcnj6*	
		CIE, NAc	*Kcnb1, Kcnc2, Kcnc4, Kcnd2, and Kcnv1*	*Kcnma1*		*Kcnj3, Kcnj6, and Kcnj13*	
		CIE, PFC	*Kcnb1, Kcnc1, and Kcnq5*	*Kcnma1*			
		HAU, NAc	*Kcna4, Kcnh2, Kcnh7, Kcnq1, Kcnq5, Kcns1, Kcns2, and Kcnv2*			*Kcnj1, Kcnj8, Kcnj11, and Kcnj16*	
		HAU, PFC	*Kcna7, Kcnc1, Kcnd2, Kcne1, and Kcnh3*	*Kcnmb2*	*Kcnk3*	*Kcnj9*	
Mouse, Hip	CIE, immediate, WB	↓EtOH v control	KCND2				[73]
Rat, OHSC	7–9 days EtOH exposure, immediate, WB	↓ EtOH (50 mM) v control	KCND2				[72]
		↓ EtOH (75 mM) v control					
Rat, LHb	Rats were injected EtOH (2g/kg i.p.) twice daily for 7 days, 24 hours, WB	Post-EtOH	KCNQ2 and KCNQ3				[76]
Rat, NAc core	IAA, 72 hours withdrawal, WB	↑ DRM v Naïve	KCNQ2				[75]
		↓ DSM v Naïve					
Mouse, extended amygdala	30-day EtOH two-bottle choice paradigm, SN RNA, expression BeadChip	↓ Post-EtOH	*Kcnq2*				[71]
HEK293	Transfected Bk subunit (BK KI), 25mM, 6 hours, IHC	↓ EtOH v Naïve, SE		KCNMA1			[77]
		↑ EtOH v Naïve, IE					
Rat, NAc core	EtOH SA 42–50 days, abstinence 3–5 weeks, WB	↓ EtOH SA v Naïve		KCNN3			[78]
Mouse, NAc core	CIE	↓ EtOH CIE v Control		KCNN3			[79]
		↓ EtOH SA v Naïve					
Mouse, CB	Chronic EtOH treatment, 4 hours, RT-PCR	↓ EtOH v control			*Kcnk1*		[80]
Mouse, VTA	Single EtOH injection IP, 4 hours, RT-PCR	↑ EtOH v control			*Kcnk13*		[50]
Mouse, NAc	Girk3 KO, injected EtOH (2g/kg i.p.), Checked DA release by microdialysis, 91–180 and 451–540 minutes	↓ Girk3 KO v WT				KCNJ9	[81]

Abbreviation: EtOH SA, alcohol self -administration; IAA intermittent access to EtOH; CIE, chronic intermittent EtOH exposure; VEC, voluntary ethanol consumption; HAU, heavy alcohol use; IP, intraperitoneal; NAc, nucleus accumbens; Hip, hippocampus; VTA, ventral tegmental area; LHb, lateral habenula; CB, cerebella; PFC, pref rontal cortex; OHSC, organotypic hippocampal slice culture; HEK293, human embryonic kidney cells 293; ↑, significantly increased; ↓, significantly decreased; WT, w ild-type; KO, knock-out; KI, knock-in; SE, surface expression; IE intracellular expression; RT-PCR, reverse transcriptase PCR; MeDIP-PCR, methylation of cytosine at CpG-PCR; IHC, immunohistochemistry; WB, w estern blot (immunoblot); DRM, detergent-resistant membrane; DSM, detergent-soluble membrane; SN, synaptoneurosomes.

**Table 3:** Potassium Channels Effected by Cocaine.

Species/Source	Experimental Design		Voltage-Gated	Inwardly Rectifying	Ref.
Rat, KCNQ2/3 agonist FLU	rats were trained	↓ FLU reduces the CPP	KCNQ2/3		[101]
		↓ FLU reduces the LA			
Mouse, *Kcnj6* WT, and K*cnj6* KO (DATCre(+):Girk2*^flox/flox^*)	Mice were trained with cocaine SA for up to 10 days then 7 day of FR3 training folowed by testing multiple cocaine doses	↑ K*cnj6* KO v WT (0.03–3.0 mg/kg) CI		*Kcnj6*	[52]
		↑ K*cnj6* KO v WT effect of genotype CT			
Mouse, WT, Kir3.2/Kcnj6 KO, and Kir3.3/Kcnj9 KO	Mice were trained with Food SA for 7 days then tested 5–9 days. Following behavioral extinction Cocaine SA began with behavior testing of multiple cocaine doses	↓ K*cnj6* KO v WT (0.125–0.5 mg/kg) APL		*Kcnj6*	[102]
		↓ K*cnj6* ko v WT (0.125–1.0 mg/kg) CI			
		↓ K*cnj6* KO v WT (0.125–1.0 mg/kg) CI mg/kg			
		↓ K*cnj9* KO v WT (0.125–0.5 mg/kg) APL		*Kcnj9*	
		↓ K*cnj9* KO v WT (0.125–1.0 mg/kg) CI			
		↓ K*cnj9* KO v WT (0.125–1.0 mg/kg) CI mg/kg			

Abbreviation: Cocaine SA, cocaine self-administration; ↑, significantly increased; ↓, significantly decreased; ALP, active lever presses; CI, cocaine infusions; CI mg/kg, cocaine intake (mg/kg); CPP, conditioned. place prefernence; LA, locomotor activation; WT, wild-type; KO, knock-out; Food SA, food self-administration; FLU, flupirtine

**Table 4:** Potassium Channels Effected by METH.

Species/Source	Experimental Design	Comparison	Voltage-Gated	Calcium-Activated	Two-Pore Domain	Inwardly Rectifying	Ref.
Rat NAc	Prior METH or Sal, METH injection, 22 days later started METH SA for 23 days, followed by 30 days abstinence, RT-PCR, WB, MeDIP-PCR	↑ SM v Sal	*Kcna1*, *Kcna3*, *Kcna5*, *Kcna6*, *Kcnb1*, and *Kcnb2*	*Kcnn1*, *Kcnn3*, *Kcnma1*, and *Kcnmb2*			[53]
		↑ MM v Sal	*Kcna4*	*Kcnn3*, *Kcnma1*, and *Kcnmb2*			
		↑ SM v Sal	*KCNA1 and KCNA3*	*KCNN1*			
		↓ SM v Sal	*KCNA1*				
Rat primary Hip neurons	METH doses, 100, 300, 900 µM, 24 hours, WB	↑ Doses v Sal	KCNB1				[115]
Rat NAc	METH SA for 20 days, then 10 days footshock, 2 hours, RT-PCR, hmC-Seq	↑ ShS v Sal	*Kcna1*, *Kcna2*, and *Kcnb2*	*Kcnn1*, *Kcnn2*, and *Kcnma1*			[55]
		↑ ShS v ShR	*Kcna1*, *Kcna2*, and *Kcnb2*	*Kcnn1*, *Kcnn2*, and *Kcnma1*			
		↑ ShS v Sal		*KCNN2 and KCNMA1*	*KCNK12*	*KCNJ2 and KCNJ3*	
		↑ShR v Sal	*KCNB2*	*KCNN2*			
		↑ ShR v ShS	*KCNA4, KCNB2, KCND3, and KCNH1*	*KCNN2*			
		↓ ShR v ShS	*KCNB2*	*KCNN2 and KCNT2*			

Abbreviation: METH SA, methamphetamine self-administration; NAc, nucleus accumbens; Hip, hippocampus; ↑, significantly increased; ↓, significantly decreased; Sal, saline; wv, weaver mutant mice; WT, wild-type; ShS, shock-sensitive; ShR, shock-resistant; SM, single saline injection followed by METH SA; MM, single METH injection followed by METH SA; hmC-Seq, hydroxymethyla-tion of cytosine at CpG-sequencing; RT-PCR, reverse. transcriptase PCR; MeDIP-PCR, methylation of cytosine at CpG-PCR; WB, western Blot (immunoblot)

**Table 5:** Potassium Channels Effected by Opioids.

Species/Source	Experimental Design		Voltage-Gated	Calcium-Activated	Inwardly Rectifying	Ref.
HEK293 stably expressing wild-type (WT) hERG channels	Whole-cell voltage clamp technique recorded IhERG from hERG-HEK cells before and after fentanyl	↓ hERG-HEK v Control	KCNH2			[61]
Mouse, Hip	Mice received escalating doses of 5, 8, 10, and 15 mg/kg morphine of saline, one week later challenged with 5 mg/kg morphine or saline. WB	↓ M+M v S+M, PSD		KCNN2		[127]
		↑ M+M v S+M, TP		KCNN2		
Mouse, Hip	WB, morphine vs CT	↓ NAc		KCNN2		[58]
		↓ NAc		KCNN3		
		↑ mPFC		KCNN3		
Mouse, VTA, Behavior	Morphine 0, 3, 10, and 30 mg/kg, *Kcnj3* KO, *Kcnj6 KO*, *Kcnj9* KO, *Kcnj9* KI VTA, LMA	↑ *Kcnj3* KO v WT 30 mg/kg			*Kcnj3*	[59]
		↑ *Kcnj6* KO v WT 10 and 30 mg/kg			*Kcnj6*	
		↓ *Kcnj9* KO v WT 10 mg/kg			*Kcnj9*	
		Normalized with *Kcnj9* KI			*Kcnj9*	
Mouse, Behavior	Oxycodone administered IT to ICV control siRNA and *Kcnj3* siRNA mice. Tail-flick was used to test antinociceptive effect MPE	↓ *Kcnj3* v Control siRNA			*Kcnj3*	[60]

Abbreviation: Hip, hippocampus; VTA, ventral tegmental area; mPFC, medial prefrontal cortex; ICV, intracerebroventricular injection; IT, intrathecal administration; HEK293, human embryonic kidney cells 293; ↑, significantly increased; ↓, significantly decreased; WT, wild-type; KO, knock-out; KI, knock-in; KD, knock-down; siRNA, small interfering RNA; WB, western blot (immunoblot); TP, total protein. homogenate; PSD, postsynaptic density/subcellular fractionation; LMA, locomotor activity; MPE, maximal possible effect; Glibenclamide, K ATP channel blocker.

## Abbreviations

AUD	Alcohol use disorder
BK	Big/large conductance
CIE	Chronic intermittent ethanol
CUD	Cocaine us disorder
DA	Dopamine
HAU	Heavy alcohol use
HIP	Hippocampus
K^+^	Potassium
K_V_	K^+^ channel voltage-gated
K_Ca_	K^+^ channel calcium-activated
K_2P_	K^+^ channel two/tandem-pore domain
K_IR_	K^+^ channel inwardly rectifying
KO	Knock-out
METH	Methamphetamine
METH SA	METH self-administration
MUD	Methamphetamine use disorder
NAc	Nucleus accumbens
OUD	Opioid use disorder
PFC	Prefrontal cortex
SK	Small conductance
SUD	Substance use disorder
VEC	Voluntary ethanol consumption
VTA	Ventral tegmental area
WT	Wild-type
